# A dataset of factors influencing consumer behavior towards bringing own shopping bags instead of using plastic bags in Vietnam

**DOI:** 10.1016/j.dib.2021.107226

**Published:** 2021-06-14

**Authors:** Thi-Phuong-Linh Nguyen

**Affiliations:** National Economics University, 207 Giai Phong, Hanoi, Vietnam

**Keywords:** Consumer behavior, Shopping bags, Plastic bags, Theory of planned behavior, Norm activation model

## Abstract

The dataset presents factors influencing consumer behavior towards bringing own shopping bags instead of using plastic bags in Vietnam. The survey was designed based on the theoretical integration model of theory of planned behavior (TPB) and norm activation model (NAM) including 8 factors, 25 items inherited from the studies. 7 other items were used to find out the respondent's characteristics, including: gender, age, educational qualification, marital status, job, number of family members and income. The questionnaires were sent in two forms: direct distribution and collection at some supermarkets; online survey via Google Docs tool to some consumers in Vietnam in November 2020. 536 valid questionnaires were collected to study factors influencing consumer behavior towards bringing own shopping bags instead of using plastic bags in Vietnam. The data set was collected as a reference source for later research on consumer behaviors to protect the environment in general and the behavior to bring own shopping bags instead of using plastic bags in particular.

## Specifications Table

**Table:** 

Subject	Consumer behavior
Specific subject area	Shopping bags, plastic bags, theory of planned behavior, norm activation model, Vietnam
Type of data	Table
How data were acquired	Questionnaire
Data format	Raw Analyzed
Parameters for data collection	Participants are consumers in Vietnam who have gone to the supermarket at least once, voluntarily participating in the survey.
Description of data collection	The data was collected in two ways: direct distribution and collection at some supermarkets; online survey via Google Docs tool to some consumers in Vietnam in November 2020. The data set includes 536 valid responses.
Data source location	Region: Asia Country: Vietnam Latitude and longitude: 21.028511, 105.804817
Data accessibility	Data with the article

## Value of the Data

• The dataset uses the integration of two TPB-NAM theories to study factors that influence consumer behavior towards bringing own shopping bags instead of using plastic bags in Vietnam.
• The dataset has collected the opinions of 536 Vietnamese consumers about a sustainable consumption behavior that is meaningful to the environment.
• The data set is a reference source for the authorities to promote sustainable consumption behavior, in particular, consumption of not using plastic when shopping at the supermarket.

## 1 Data Description

Sustainable consumption is the purchase, use and disposal of products in a manner that reduces damage to the environment [1,2]. While consumers may understand the importance of sustainability and are willing to accept a greener option when surveyed, the actual adoption rate is surprisingly low [1,3]. In fact, plastic bags are no exception, although most consumers are aware that plastic bags pose a danger to the environment, but not many consumers bring own shopping bags instead of using plastic bags [4,5]. The use or not to use plastic bags when shopping is related to ethical consumer behavior. Ethical consumers regularly buy and use environmentally friendly products [6]. Therefore, behavior towards bringing own shopping bags instead of using plastic bags should be studied in two approaches: an approach based on a common behavior perspective, one based on an ethical perspective [1,5]. Therefore, the data set gathered consumer opinion based on 8 factors from the TPB-NAM integration model to study about the behavior of bringing own shopping bags instead of using plastic bags, in which, TPB explores common consumer behavior while NAM emphasizes on ethical views in consumption. In particular, TPB and NAM have been integrated and used in a number of studies on consumer behavior [7], [8], [9], [10], [11] but never used to study the behavior of bringing own shopping bags instead of using plastic bags.

The data set was collected through a 2-part survey: the first part explores the respondents' characteristics including: gender, age, educational qualification, marital status, job, number of family members and income (Table 1); the second part explores respondents' consent to statements related to factors influencing consumer behavior towards bringing own shopping bags instead of using plastic bags in Vietnam (Table 2); Table 3 shows more detailed results between the variables.

**Table 1:** Respondents’ characteristics.

*Characteristics*	*N*	*%*
*Gender (RC1)*	536	100.00
Male	151	28.2
Female	384	71.6
*Age (RC2)*	536	100.00
Under 20	56	10.45
From 20 to 29	208	38.81
From 30 to 39	145	27.05
From 40 to 49	48	8.96
From 50 to 59	64	11.94
Over 60	15	2.80
*Educational qualification (RC3)*	536	100.00
High School Graduation	114	21.27
College/University Graduation	339	63.25
Master/PhD graduation	47	8.77
Others	36	6.72
*Job (RC4)*	536	100.00
Student	98	18.28
Business staff	114	21.27
State employee	176	32.84
Housewife	79	14.74
Freelancer	69	12.87
*Marital status (RC5)*	536	100.00
Single	235	43.84
Married	249	46.46
Divorce	36	6.72
Other	16	2.99
*Number of family members (RC6)*	536	100.00
1	41	7.65
From 2 to 4	367	68.47
Upper 4	128	23.88
*Income (RC7)*	536	100.00
Under 6 million VND	77	14.37
From 6 million to 10 million VND	139	25.93
From 10 million to 20 million VND	184	34.33
From 20 million to 30 million VND	65	12.13
From 30 million to 40 million VND	41	7.65
Upper 40 million VND	30	5.60

**Table 2:** Descriptive results of participants’ responses.

Variables		N	Min	Max	Mean	Std. Deviation
**Behavior (BE) *(Cronbach's Alpha = 0.916)***						
BE1	If plastic bags given at cash registers were not free, I would use fewer plastic bags.	536	1	5	4.076	0.9800
BE2	If supermarkets offered discounts to shoppers who brought their own cloth bags, I would use fewer plastic bags.	536	1	5	4.226	0.9086
BE3	I usually bring my own bags when shopping	536	1	5	3.959	0.8702
***Intention (IN) (Cronbach's Alpha = 0.841)***						
IN1	I will buy fabric bag products to use when shopping.	536	1	5	3.763	0.8371
IN2	I plan to continue with the choice of buying fabric bag products for future shopping.	536	1	5	3.823	0.8136
IN3	I will recommend for everyone to use the eco-friendly fabric bag.	536	1	5	3.866	0.8161
***Attitude (AT) (Cronbach's Alpha = 0.797)***						
AT1	I like to take advantage of shopping situations to get free plastic bags.	536	1	5	4.209	0.9339
AT2	It is worthwhile to bring my own bag(s) to shopping.	536	1	5	4.300	0.8589
AT3	It is stupid for me to hold shopping items with my bare hands.	536	1	5	3.978	0.7945
***Subject norms (SN) (Cronbach's Alpha = 0.858)***						
SN1	The people who influence my behavior think that I should bring a cloth bag when I go shopping.	536	1	5	3.584	0.9315
SN2	My close friends think that I should use cloth bags when shopping.	536	1	5	3.539	0.9365
SN3	Most of the people important to me think that I should bring cloth bags when shopping.	536	1	5	3.444	0.9276
***Perceived behavioral control (PBC) (Cronbach's Alpha = 0.821)***						
PBC1	I will use cloth bag when I go shopping although friends advise me not to use it due to inconvenience.	536	1	5	3.815	0.8302
PBC2	I have complete control over the use of cloth bags when shopping.	536	1	5	3.651	0.9148
PBC3	I can afford to buy fabric bag products to use when shopping.	536	1	5	3.849	0.9558
***Awareness of consequences (AC) (Cronbach's Alpha = 0.952)***						
AC1	Plastic bags damage the environment.	536	1	5	4.547	0.8827
AC2	Plastic bags increase the risk of cancer.	536	1	5	4.466	0.9042
AC3	Plastic bags harm living beings (animals) on land.	536	1	5	4.522	0.8962
AC4	Plastic bag wastes emit toxic gases into the air.	536	1	5	4.451	0.9158
***Ascription of responsibility (AR) (Cronbach's Alpha = 0.880)***						
AR1	I have an obligation to bring cloth bags when shopping morally.	536	1	5	3.817	0.9707
AR2	Carrying a cloth bag with you when shopping is ethical.	536	1	5	3.903	0.9462
AR3	Walking behavior when I carry cloth bags is ethically correct.	536	1	5	3.946	0.9347
***Personal norm (PN) (Cronbach's Alpha = 0.908)***						
PN1	Every citizen has the obligation to avoid using plastic bags.	536	1	5	4.151	0.9320
PN2	I feel obliged to comply with the government's plastic bag restriction.	536	1	5	4.144	0.8689
PN3	Unless many people comply with the restriction, I do not have the responsibility to comply.	536	1	5	4.032	0.8117

**Table 3:** Correlations between variables and customer behavior towards bringing own shopping bags instead of using plastic bags in Vietnam.

		*Behavior*		
Variable		BE1	BE2	BE3
***Respondent characteristic***				
RC1	Gender	0.347⁎⁎	0.367⁎⁎	0.397⁎⁎
RC2	Age	0.273⁎⁎	0.285⁎⁎	0.303⁎⁎
RC3	Educational qualification	0.538⁎⁎	0.554⁎⁎	0.577⁎⁎
RC4	Marital status	0.081	0.050	0.047
RC5	Job	0.059	0.076	0.101*
RC6	Number of family members	0.045	0.053	0.040
RC7	Income	0.053	0.066	0.060
***Intention***				
IN1	I will buy fabric bag products to use when shopping.	0.298⁎⁎	0.346⁎⁎	0.400⁎⁎
IN2	I plan to continue with the choice of buying fabric bag products for future shopping.	0.341⁎⁎	0.383⁎⁎	0.452⁎⁎
IN3	I will recommend for everyone to use the eco-friendly fabric bag.	0.291⁎⁎	0.386⁎⁎	0.400⁎⁎
***Attitude***				
AT1	I like to take advantage of shopping situations to get free plastic bags.	0.025	0.035	0.022
AT2	It is worthwhile to bring my own bag(s) to shopping.	0.022	0.028	0.029
AT3	It is stupid for me to hold shopping items with my bare hands.	0.026	0.067	0.069
***Subject norms***				
SN1	The people who influence my behavior think that I should bring a cloth bag when I go shopping.	0.047	0.080	0.122⁎⁎
SN2	My close friends think that I should use cloth bags when shopping.	0.018	0.054	0.094*
SN3	Most of the people important to me think that I should bring cloth bags when shopping.	−0.005	0.014	0.085*
***Perceived behavioral control***				
PBC1	I will use cloth bag when I go shopping although friends advise me not to use it due to inconvenience.	0.001	0.045	0.021
PBC2	I have complete control over the use of cloth bags when shopping.	−0.020	0.014	−0.023
PBC3	I can afford to buy fabric bag products to use when shopping.	0.016	0.072	0.042
***Awareness of consequences***				
AC1	Plastic bags damage the environment.	−0.007	−0.014	−0.002
AC2	Plastic bags increase the risk of cancer.	0.021	0.015	0.029
AC3	Plastic bags harm living beings (animals) on land.	0.016	0.013	0.006
AC4	Plastic bag wastes emit toxic gases into the air.	0.028	−0.004	0.035
***Ascription of responsibility***				
AR1	I have an obligation to bring cloth bags when shopping morally.	0.048	0.087*	0.100*
AR2	Carrying a cloth bag with you when shopping is ethical.	−0.008	0.030	0.043
AR3	Walking behavior when I carry cloth bags is ethically correct.	0.019	0.067	0.052
***Personal norm***				
PN1	Every citizen has the obligation to avoid using plastic bags.	0.354⁎⁎	0.386⁎⁎	0.397⁎⁎
PN2	I feel obliged to comply with the government's plastic bag restriction.	0.384⁎⁎	0.409⁎⁎	0.431⁎⁎
PN3	Unless many people comply with the restriction, I do not have the responsibility to comply.	0.300⁎⁎	0.325⁎⁎	0.343⁎⁎

⁎⁎ Correlation is significant at the 0.01 level (2-tailed).

⁎ Correlation is significant at the 0.05 level (2-tailed).

The survey was sent to consumers through two forms: direct distribution and collection at some supermarkets; online survey via Google Docs tool to some consumers in Vietnam in November 2020. In the form of direct distribution at some supermarkets, the author observed and instructed the answer, each consumer spent about 15 min completing the survey. For the form of online survey via Google Docs tool, the author gathered emails of individuals in some agencies and organizations operating in Vietnam, then sent the survey questionnaires link directly via their email. The results obtained a total of 536 valid survey questionnaires in both forms. In which, in the form of survey at supermarkets, the author obtained 211 valid questionnaires out of the total of 235 questionnaires, reaching the rate of 89.8%; in the form of online surveys, the author obtained 325 valid questionnaires out of 600 emails sending survey questionnaire links to consumers, reaching the rate of 54.2%. The questionnaire and answers were shown in the supplementary files.

The dataset includes: the respondent's characteristics (Table 1) and 8 factors: (1) behavior; (2) intention; (3) attitude; (4) subject norms; (5) perceived behavioral control; (6) awareness of consequences; (7) ascription of responsibility; (8) personal norm (Table 2).

## 2 Experimental Design, Materials and Methods

The survey is done simultaneously by both direct and online forms in November 2020. With the form of direct distribution and collection at some supermarkets, the author has listed top 10 supermarkets in two big cities, Hanoi and Ho Chi Minh, with the most number of shoppers in Vietnam, then with the support of collaborators, the author conducted the survey at 9 am, 10 am every weekend at the exit of the supermarkets. With the form of online survey via Google Docs, the author makes a list of several enterprises that publish their employees' email addresses on the company's official website and randomly selects 50 enterprises with diverse business sectors, then sends the survey link to these people. Each survey participant received a prize number and 10 lucky people, randomly selected, received a supermarket shopping voucher.

The survey was designed with 32 items, of which 7 were about respondents’ characteristics, the remaining 25 items, are designed on a 5-point Likert scale (1: Strongly disagree; 2: Disagree; 3: Neutral; 4: Agree; 5: Strongly agree), focus on 8 factors: behavior, intention, attitude, subject norms, perceived behavioral control, awareness of consequences, ascription of responsibility and personal norm. All items in the survey are inherited from previous studies [4,5,12,13]. The questionnaire is only valid when it meets two conditions: firstly, answering all questions; secondly answering two inversion questions AT1 and PN3 in accordance with results of other questions. After discarding the invalid questionnaires, the final data set contained 536 questionnaires. All respondents' responses were encrypted and imported into Excel software before importing to SPSS 22.

Based on the data set, further studies can study the relationship between factors in the TPB-NAM integration model or separate each theory to find factors influencing consumer intention and behavior towards bringing own shopping bags instead of using plastic bags in Vietnam.

## Ethics Statement

The authors kept to all ethical concerns during the data gathering process. The authors got the consent of the response when conducting surveys. Research has been conducted in an environment that does not require ethical approval for survey studies.

## Declaration of Competing Interest

The authors declare that they have no known competing financial interests or personal relationships which have, or could be perceived to have, influenced the work reported in this article.

## Appendix Supplementary materials

Image, application 1

Image, application 2
