# Supplementary material for: A dataset of factors influencing consumer behavior towards bringing own shopping bags instead of using plastic bags in Vietnam
Source: Data Brief. 2021 Jun 14;37:107226. doi: 10.1016/j.dib.2021.107226 (PMC8220313; doi:10.1016/j.dib.2021.107226)
Supplement: Supplementary file 1 [file mmc1.docx]

**QUESTIONNAIRE**

**A dataset of factors influencing consumer behavior towards bringing own shopping bags instead of using plastic bags in Vietnam**

**PART 1.** Respondents’ characteristics

***1) Gender***

| _1_ Male |
| --- |
| _2_ Female |

***2) Age***

| _1_ Under 20 |
| --- |
| _2_ From 20 to 29 |
| _3_ From 30 to 39 |
| _4_ From 40 to 49 |
| _4_ From 50 to 59 |
| _5_ Over 60 |

***3) Educational qualification***

| _1_ High School Graduation |
| --- |
| _2_ College/University Graduation |
| _3_ Master/PhD graduation |
| _4_ Others |

***4) Job***

| _1_ Student |
| --- |
| _2_ Business staff |
| _3_ State employee |
| _4_ Housewife |
| _5_ Freelancer |

***5) Marital status***

| _1_ Single |
| --- |
| _2_ Married |
| _3_ Divorce |
| _4_ Other |

***6) Number of family members***

| _1_ 1 |
| --- |
| _2_ From 2 to 4 |
| _3_ Upper 4 |

***7) Income***

| _1_ Under 6 million VND |
| --- |
| _2_ From 6 million – 10 million VND |
| _3_ From 10 million – 20 million VND |
| _4_ From 20 million – 30 million VND |
| _5_ From 30 million – 40 million VND |
| _6_ Upper 40 million VND |

**PART 2.** Please indicate your level of agreement to the following statements by adding ⌧ or 🗹 to the numbers 1 to 5. Correspondingly: 1 - strongly disagree, 2 - disagree, 3 - Neutral, 4 - agree, 5 - strongly agree.

| **Variables** | | **1** | **2** | **3** | **4** | **5** |
| --- | --- | --- | --- | --- | --- | --- |
| ***Behavior (BE)*** | | | | | | |
| BE1 | If plastic bags given at cash registers were not free, I would use fewer plastic bags. | ① | ② | ③ | ④ | ⑤ |
| BE2 | If supermarkets offered discounts to shoppers who brought their own cloth bags, I would use fewer plastic bags. | ① | ② | ③ | ④ | ⑤ |
| BE3 | I usually bring my own bags when shopping | ① | ② | ③ | ④ | ⑤ |
| ***Intention (IN)*** | | | | | | |
| IN1 | I will buy fabric bag products to use when shopping. | ① | ② | ③ | ④ | ⑤ |
| IN2 | I plan to continue with the choice of buying fabric bag products for future shopping. | ① | ② | ③ | ④ | ⑤ |
| IN3 | I will recommend for everyone to use the eco-friendly fabric bag. | ① | ② | ③ | ④ | ⑤ |
| ***Attitude (AT)*** | | | | | | |
| AT1 | I like to take advantage of shopping situations to get free plastic bags. | ① | ② | ③ | ④ | ⑤ |
| AT2 | It is worthwhile to bring my own bag(s) to shopping. | ① | ② | ③ | ④ | ⑤ |
| AT3 | It is stupid for me to hold shopping items with my bare hands. | ① | ② | ③ | ④ | ⑤ |
| ***Subject norms*** | | | | | | |
| SN1 | The people who influence my behavior think that I should bring a cloth bag when I go shopping. | ① | ② | ③ | ④ | ⑤ |
| SN2 | My close friends think that I should use cloth bags when shopping. | ① | ② | ③ | ④ | ⑤ |
| SN3 | Most of the people important to me think that I should bring cloth bags when shopping. | ① | ② | ③ | ④ | ⑤ |
| ***Perceived behavioral control (PBC)*** | | | | | | |
| PBC1 | I will use cloth bag when I go shopping although friends advise me not to use it due to inconvenience. | ① | ② | ③ | ④ | ⑤ |
| PBC2 | I have complete control over the use of cloth bags when shopping. | ① | ② | ③ | ④ | ⑤ |
| PBC3 | I can afford to buy fabric bag products to use when shopping. | ① | ② | ③ | ④ | ⑤ |
| ***Awareness of consequences (AC)*** | | | | | | |
| AC1 | Plastic bags damage the environment. | ① | ② | ③ | ④ | ⑤ |
| AC2 | Plastic bags increase the risk of cancer. | ① | ② | ③ | ④ | ⑤ |
| AC3 | Plastic bags harm living beings (animals) on land. | ① | ② | ③ | ④ | ⑤ |
| AC4 | Plastic bag wastes emit toxic gases into the air. | ① | ② | ③ | ④ | ⑤ |
| ***Ascription of responsibility (AR)*** | | | | | | |
| AR1 | I have an obligation to bring cloth bags when shopping morally. | ① | ② | ③ | ④ | ⑤ |
| AR2 | Carrying a cloth bag with you when shopping is ethical. | ① | ② | ③ | ④ | ⑤ |
| AR3 | Walking behavior when I carry cloth bags is ethically correct. | ① | ② | ③ | ④ | ⑤ |
| ***Personal norm (PN)*** | | | | | | |
| PN1 | Every citizen has the obligation to avoid using plastic bags. | ① | ② | ③ | ④ | ⑤ |
| PN2 | I feel obliged to comply with the government’s plastic bag restriction. | ① | ② | ③ | ④ | ⑤ |
| PN3 | Unless many people comply with the restriction, I don’t have the responsibility to comply. | ① | ② | ③ | ④ | ⑤ |
